# Low preoperative psoas muscle mass index is a risk factor for distal cholangiocarcinoma recurrence after pancreatoduodenectomy: a retrospective analysis

**DOI:** 10.1186/s12957-022-02627-w

**Published:** 2022-06-02

**Authors:** Saori Umezawa, Shinjiro Kobayashi, Takehito Otsubo

**Affiliations:** Division of Gastroenterological and General Surgery, St. Marianna University School of Medicine, 2-16-1 Sugao, Miyamae-ku, Kawasaki, Kanagawa 216-8511 Japan

**Keywords:** Distal cholangiocarcinoma, Pancreatoduodenectomy, Psoas muscle mass index, Sarcopenia

## Abstract

**Background:**

This study aimed to investigate whether preoperative muscle mass is associated with the recurrence of distal cholangiocarcinoma after pancreatoduodenectomy (PD).

**Methods:**

We retrospectively examined 88 patients who had undergone PD for distal cholangiocarcinoma. The preoperative psoas muscle mass index (PMI) was measured using computed tomography as an index of muscle mass. We performed multivariate analysis of factors influencing early recurrence and developed a prognostic survival model using independent risk factors for recurrence.

**Results:**

The cut-off PMI values for recurrence within 1 year of surgery, determined from the receiver operating characteristic curve, were 5.90 cm^2^/m^2^ in males and 3.98 cm^2^/m^2^ in females. Multivariate analysis of effects associated with early recurrence within 1 year indicated that low PMI (odds ratio [OR] 9.322; 95% confidence interval [CI] 2.832 − 30.678; *p* = 0.0002) and lymph node metastasis (OR 5.474; 95% CI 1.620 − 18.497; *p* = 0.0062) were independent risk factors, and the median recurrence-free survival (RFS) of the low and high PMI groups were 21.6 and 81.0 months, respectively (*p* = 0.0214). The median RFS for zero, one, and two risk factors of low PMI and lymph node metastasis were as follows: zero variables, median not reached; one variable, 15.3 months; two variables: 6 months.

**Conclusions:**

Low preoperative PMI may be a risk factor for distal cholangiocarcinoma recurrence after PD.

**Trial registration:**

The Institutional Review Board of St. Marianna University School of Medicine approved this study prior to commencement of data collection and analysis on October 9, 2020 (IRB no. 5006) and waived the informed consent requirement.

**Supplementary Information:**

The online version contains supplementary material available at 10.1186/s12957-022-02627-w.

## Background

Since Rosenberg proposed the term “sarcopenia” for age-related skeletal muscle dysfunction in 1997 [1], the research on the association between declines in skeletal muscle mass, muscle strength, and physical function and worsened outcomes of various diseases has progressed [2, 3]. In addition, sarcopenia has recently been reported to be a risk factor for postoperative complications of various gastrointestinal cancer surgeries [4–6]. An increasing number of reports have also shown that sarcopenia affects gastrointestinal cancer prognosis [7–10].

Although there have been several reports on the association between muscle mass and prognosis in biliary tract cancer, these studies focus only on intrahepatic [11] or hilar cholangiocarcinomas [12] or analyze gallbladder carcinoma, intrahepatic cholangiocarcinoma, hilar cholangiocarcinoma, and distal cholangiocarcinoma as biliary tract cancer [13]. There are few studies that have examined the correlation between muscle mass and prognosis only for distal cholangiocarcinoma [14]. The standard procedure for distal cholangiocarcinoma is pancreatoduodenectomy (PD), but PD is highly invasive, and the risk of the surgery itself is high for patients with low muscle mass [15]. If low muscle mass is a prognostic factor in addition to the high risk of the surgery itself, muscle mass may be a very important issue for patients with distal cholangiocarcinoma.

On the other hand, there have been studies on the predictors of postoperative recurrence of distal cholangiocarcinoma; however, few studies have focused on the predictors of its early recurrence [16]. The prognosis for gastrointestinal cancer patients with early recurrence is extremely poor, and surgery and adjuvant chemotherapy alone may not be sufficient for the treatment of patients prone to early recurrence [17, 18]. If early recurrence is predicted, the treatment strategy should be considered, including the use of adjuvant chemotherapy even before surgery. Therefore, the prediction of early recurrence is an important issue for prolonging the prognosis of cholangiocarcinoma. In this study, we investigated the relationship between preoperative muscle mass and early recurrence in patients with distal cholangiocarcinoma who underwent PD.

## Methods

We retrospectively examined the medical records of distal cholangiocarcinoma patients who had undergone PD at St. Marianna University of Medicine between January 2006 and April 2020 and whose diagnosis was confirmed by postoperative pathological examination.

### Surgical procedures

In jaundiced patients (the serum level of total bilirubin was > 4 mg/mL), endoscopic nasal biliary drainage, endoscopic retrograde biliary drainage, or percutaneous transhepatic catheter drainage was performed to reduce yellowing until the serum total bilirubin was < 4 mg/mL preoperatively.

For either procedure, lymph nodes from the following areas were removed: hepatoduodenal ligament (#12 h, #12a, #12b, #12p), circumferentially around the common hepatic artery (#8a, #8p), and the right-half circumference of the superior mesenteric artery (#14p, #14d). In all cases, the lymph nodes around the celiac artery (#9) were not dissected. When the bile duct was dissected, the bile duct on the hepatic side was clamped, and that on the resected side was ligated to prevent bile from leaking into the abdominal cavity. The clamping of the bile duct on the hepatic side was continued until the bile duct jejunum was subsequently reconstructed. Bile duct transects were submitted for intraoperative rapid pathology in all cases, and if they were positive for cancer, additional resections were performed until they became negative for cancer. If it was difficult to distinguish inflammation from cancer or if cancer was suspected only in the superficial layer, and if the bile duct could not be resected any further without a combined resection of the liver, no additional resection was performed. All patients underwent modified Child reconstruction. Pancreatic anastomosis after PD was performed through duct-to-mucosa and end-to-side pancreatojejunostomy in all patients. Cholangiojejunostomy was performed via end-to-side anastomosis in all patients. Postoperatively, digestive enzyme supplementation was initiated, and at the same time, oral intake was started.

### Retrospective chart review

**Fig. 1:**
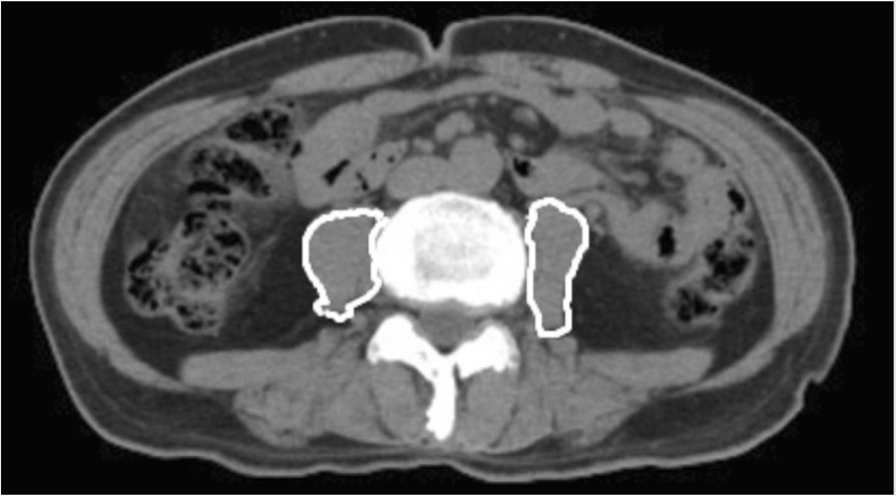
Cross-sectional computed tomography image at the third lumbar vertebral level.The areas of the bilateral psoas muscle were measured by manual tracing

We used 5-mm preoperative images constructed with 64-row computed tomography (CT) (Aquilion, Canon Medical Systems) using 0.5-mm-thick imaging slices to measure psoas muscle mass index (PMI). CT images were taken preoperatively. The median date of imaging was 18 days preoperatively (interquartile range [IQR] 10 − 28 days). The cross-sectional areas of the bilateral psoas major muscles were measured by manually tracing CT images at the third lumbar vertebral level (Fig. 1), and the PMI was calculated as follows:

$$\mathrm{PMI}\;(\mathrm{cm}^2/\mathrm m^2)\:=\left(\mathrm{cross}-\mathrm{sectional}\;\mathrm{area}\;\mathrm{of}\;\mathrm{the}\;\mathrm{right}\;\mathrm{psoas}\;\mathrm{major}\;\mathrm{muscle}\:+\:\mathrm{cross}-\mathrm{sectional}\;\mathrm{area}\;\mathrm{of}\;\mathrm{the}\;\mathrm{left}\;\mathrm{psoas}\;\mathrm{major}\;\mathrm{muscle}\right)\:/\mathrm{height}^2$$

Cases that recurred within 1 year were defined as early recurrence, and background factors in the early recurrence and non-early recurrence groups were compared. Then, preoperative PMI cut-off values to establish the risk of early recurrence were defined using the receiver operating characteristic (ROC) curve. Next, we performed a multivariate analysis of factors that influence early recurrence. The factors analyzed were age, sex, presence of preoperative cholangitis, bilirubin level prior to reduction of yellowing, presence of biliary drainage, preoperative PMI, preoperative neutrophil-to-lymphocyte ratio (NLR), preoperative platelet-to-lymphocyte ratio (PLR), preoperative prognostic nutritional index (PNI), preoperative modified Glasgow Prognostic Score (mGPS), preoperative carbohydrate antigen 19–9, preoperative C-reactive protein (CRP), operation time, blood loss volume, postoperative pathological examination for tumor size, differentiation, depth, lymphatic invasion, microscopic venous invasion, perineural invasion, pancreatic invasion, and lymph node metastasis, resectability, postoperative complications, and presence or absence of adjuvant chemotherapy. The definition of elderly in Japan was adopted, and the cutoff value for age was 75 years or older. The cut-off values of NLR, PLR [19, 20], and PNI [21, 22] were those previously reported, and the cut-off values of mGPS were used for CRP. The cut-off values for operative time and blood loss volume were calculated from the median values.

Resectability was defined as R1 if invasive cancer was found within 5 mm of the bile duct wall edge and dissecting surface. Intraepithelial carcinoma was defined as R0.

In addition, we examined prognostic survival models for the independent risk factors of early recurrence obtained through multivariate analysis.

### Prognostic survival model

For staging, postoperative histopathological diagnosis was classified according to the Union for International Cancer Control 7th edition [23]; postoperative complications were classified according to the Clavien-Dindo classification [24]. Contrast-enhanced CT was performed every 3 months postoperatively, and recurrence was judged by both a radiologist and surgeon based on findings that included the appearance of liver or lung tumor, lymphadenopathy, and ascites.

The Institutional Review Board of St. Marianna University School of Medicine approved this study prior to the commencement of data collection and analysis (IRB no. 5006) and waived the informed consent requirement.

### Statistical analyses

There were no missing data in this study. All statistical computations were performed using JMP 14 (SAS Institute Inc., Cary, NC). Descriptive data are reported as medians and IQR, or counts and proportions, depending on the data type. Recurrence-free survival (RFS) was estimated using the Kaplan–Meier method and compared using the log-rank test. Multivariate logistic regression analysis was performed using factors with *p* values < 0.05 in the univariate analysis. All statistical analyses were two-tailed, and *p* values < 0.05 were considered statistically significant.

## Results

### Baseline characteristics

Between January 2006 and April 2020, there were 101 cases of distal cholangiocarcinoma diagnosed by postoperative pathological examination after PD at St. Marianna University of Medicine. Among the 101 patients, 88 were included in this study. Five patients diagnosed with adenosquamous cell carcinoma by histopathological examination, seven who underwent hepatopancreatoduodenectomy, and one with a serious postoperative complication (CD5) were excluded (Additional file 1: Table 1). The median observation period for eligible patients was 34.7 months (IQR 16.3 − 60.2 months).

Forty-six of 88 patients received postoperative adjuvant chemotherapy. Postoperative adjuvant chemotherapy consisted of gemcitabine monotherapy in 34 patients and S-1 monotherapy in 12 patients.

Following recurrence, chemotherapy or palliative treatment was selected after discussing the case with the patient.

### Comparison between the early recurrence and non-early recurrence groups

The 37 patients in the early recurrence group with recurrence within 1 year and the 51 patients in the non-early recurrence group were compared based on each parameter (Additional file 2: Table 2).

PMI values in the early recurrence and non-early recurrence groups were 5.07 cm^2^/m^2^ (IQR 4.40 − 6.13 cm^2^/m^2^) and 6.21 cm^2^/m^2^ (IQR 5.47 − 7.14 cm^2^/m^2^) (*p* = 0.0126*) and 3.62 cm^2^/m^2^ (IQR 3.0 − 4.4 cm^2^/m^2^) and 4.26 cm^2^/m^2^ (IQR 3.98 − 4.56 cm^2^/m^2^) (*p* = 0.2926) for males and females, respectively. Significant differences were observed in differentiation and lymph node metastasis, but no significant differences were observed in age, sex, preoperative status, blood test, or pathological factors.

### Cut-off values for PMI in early recurrence cases

**Fig. 2:**
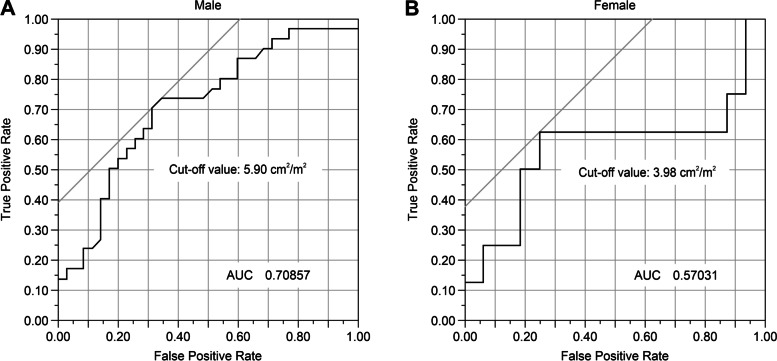
Receiver operating characteristic (ROC) curves were created using psoas muscle mass index (PMI) values of the early recurrence and non-early recurrence groups

ROC curves were plotted for PMI of both males and females, and the optimal cut-off value for early recurrence was obtained via the Youden Index. The areas under the ROC curve for PMI were 0.70857 for males and 0.57031 for females, and a convex curve was drawn on the upper left side. The optimal cut-off values for early recurrence were 5.90 and 3.98 cm^2^/m^2^ for males and females, respectively (Fig. 2). PMI cut-off was used to group the patients into low and high PMI groups (Additional file 3: Table 3), and there were no significant differences in background factors between the two groups.

### Risk factors for recurrence

Multivariate analysis of the effects associated with early recurrence indicated that low PMI (males < 5.90 cm^2^/m^2^; females < 3.98 cm^2^/m^2^) (odds ratio [OR] 9.322; 95% confidence interval [CI] 2.832 − 30.67; *p* = 0.0002)and lymph node metastasis (OR 5.474; 95% CI 1.620 − 18.49; *p* = 0.0062) were independent risk factors (Additional file 4: Table 4).

### Development of a prognostic survival model using low PMI and lymph node metastasis

**Fig. 3:**
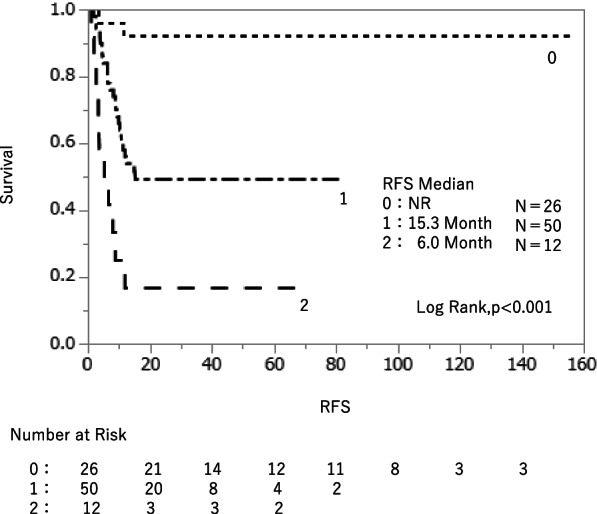
A prognostic survival model using low PMI and lymph node metastasis was created based on the regression coefficients of the multivariable model. Patients with zero prognostic factors had the longest RFS, and RFS shortened significantly as more factors were added

A prognostic survival model using low PMI and lymph node metastasis was developed based on the regression coefficients of the multivariable model. The RFS was examined according to the two independent risk factors for recurrence, including low PMI and lymph node metastasis. Patients who did not have any of the two recurrence risk factors did not reach the median RFS, those who had one of the two recurrence risk factors had a median RFS of 15.3 months, and those who had the two recurrence risk factors had a median RFS of 6.0 months (*p* < 0.001) (Fig. 3).

## Discussion

In this study, we established the cut-off PMI values to determine the risk of early recurrence of distal cholangiocarcinoma within 1 year after PD; the cut-off PMI was 5.90 cm^2^/m^2^ for males and 3.98 cm^2^/m^2^ for females. These values approximate the cut-off values (6.36 cm^2^/m^2^ for males and 3.92 cm^2^/m^2^ for females) that defined the low muscle mass of 541 Japanese living donor liver transplant recipients in previous studies [25, 26]. Although the cut-off values themselves vary by race and need to be established for each country and region, the cut-off value defining low muscle mass and the cut-off value for muscle mass as a risk factor for recurrence were similar in the same race.

In addition, preoperative low PMI and lymph node metastasis were independent risk factors for early recurrence; the median RFS for patients without any of these factors was not reached, whereas the median RFS for patients with both the factors was significantly shorter (6.0 months). In this study, only RFS was examined for prognosis, whereas overall survival was not examined due to the notion that the presence or absence of treatment after recurrence and the content of treatment would lead to bias.

In a study of 117 patients with biliary tract cancer, Chakedis J et al. [13] reported that the more the prognostic factors among low albumin, low PMI, and low psoas muscle density a patient had, the worse his/her prognosis. This report also showed that low PMI was an independent prognostic factor, but it analyzed gallbladder cancer, intrahepatic cholangiocarcinoma, hilar cholangiocarcinoma, and distal cholangiocarcinoma together as biliary tract cancer; it did not take into account the differences in the grade of carcinoma or the degree of invasiveness of the surgery. In this study, we focused on patients with distal cholangiocarcinoma who underwent PD and found that low PMI was an independent prognostic factor in distal cholangiocarcinoma. R0 resection, lymph node metastasis, nerve invasion [27, 28], T stage, and tumor size [29] have been thus far reported as prognostic factors for distal cholangiocarcinoma, but low muscle mass is expected to become an established prognostic factor through further high-quality and large-scale studies in the future.

There have been numerous reports of studies on low muscle mass and the clinical course of cancer. In pancreatic cancer, it has been reported that surgical complications are more common in patients with low muscle mass, which also affects long-term survival [30], and that PMI at recurrence correlates with prognosis in patients with recurrent disease [31].

In addition, low muscle mass has been reported to be a poor prognostic factor in hepatocellular cancer, gastric cancer, colon cancer, ovarian cancer, and lung cancer [32–36]. Matsunaga et al. analyzed 67 patients with recurrent gastric cancer and found that the incidence of chemotherapy side effects was significantly higher in the low skeletal muscle mass index (SMI) group compared with the high SMI group (11 patients; 32.4% vs. 21 patients; 63.6%, *p* = 0.010), and the median survival rate was also affected (17.8 vs. 15.8 months; *p* = 0.034) [37].

Although low muscle mass has been reported to be associated with postoperative complications such as liver resection, gastrectomy, and radical cystectomy for bladder cancer [38–40], there was no difference in the frequency or severity of complications between the low PMI and high PMI groups in the cases studied here. Lee et al., retrospectively analyzed 214 patients with obstructive colorectal cancer. Pre-sarcopenia (71 patients; 33.2%) had a negative oncologic impact in both disease-free survival (DFS) and overall survival (OS), (hazard ratio [HR] = 1.86, 95% CI 1.04–3.13, *p* = 0.037, and HR = 1.92, CI 1.02–3.60, *p* = 0.043, respectively). On the other hands, there were no significant differences in minor (11.8%vs. 12.5%) or major postoperative complications (5.6%vs. 6.9%) observed between non-pre-sarcopenia and pre-sarcopenia patients [41].

Exactly how muscle mass affects cancer prognosis has not been studied in detail; however, loss of muscle mass may be associated with cancer recurrence through several mechanisms. Aoi et al. [42] reported that a type of myokine secreted by muscle tissues suppresses the development of colon tumors by increasing apoptosis. Furthermore, an association between myokine and immune system cells, such as natural killer cells, has been reported [43]. Therefore, loss of muscle mass may decrease myokine production, resulting in an increase in cancer recurrence risk.

Although it is possible to intervene preoperatively for low PMI and low PNI, there are currently no established guidelines for preoperative nutritional guidance and exercise therapy intervention for patients with low muscle mass. Moreover, many of the diseases targeted in gastrointestinal surgery are malignant and often do not offer sufficient time before surgery. Nevertheless, the need for preoperative intervention for low muscle mass is increasing [44]. Yamamoto et al. [45] reported that preoperative exercise and nutritional guidance for elderly gastric cancer patients diagnosed with sarcopenia resulted in a significant increase in handgrip strength and an increasing trend in walking speed and SMI. The median duration of preoperative intervention in this study was 16 days (7–26 days), and the sarcopenia parameters improved even in a relatively short time, suggesting that intervention with exercise and nutritional guidance is desirable whenever possible, even if there is no time to spare before surgery. Preoperative adjuvant chemotherapy may be necessary, especially if low muscle mass is a risk factor for early recurrence, as a surgery-first treatment strategy may not be sufficient in the first place. However, since there are reports that a decrease in muscle mass during neoadjuvant chemotherapy is associated with a worse prognosis [46], it is necessary to maintain muscle mass by strengthening nutrition and exercise therapy during preoperative adjuvant chemotherapy. It is also reported that a decrease in muscle mass in the acute postoperative period is associated with a worse prognosis [47], so it is important to maintain muscle mass through postoperative nutritional management and exercise therapy, including ambulation promotion.

Although CT and magnetic resonance imaging (MRI) are used as the gold standard for measuring muscle mass according to the European Working Group on Sarcopenia in Older People [48], MRI is not frequently used due to its high cost and the time needed for analysis. Compared to dual energy X-ray absorptiometry and bioelectrical impedance analysis, using CT to measure muscle mass has the disadvantages of higher examination costs and radiation exposure to patients. Nevertheless, CT is required to diagnose and evaluate treatment strategies for patients with cancer. In this study, we measured the skeletal muscle mass at the level of the third lumbar vertebra using CT performed for cancer treatment. Skeletal muscle mass at the third lumbar vertebral level is considered to correlate with systemic skeletal muscle mass [49], and the value obtained by dividing the skeletal muscle cross-sectional area at this level by the square of the height is frequently used as the third lumbar vertebra skeletal muscle mass index (L3SMI) [50, 51]. PMI, which measures muscle mass based only on the cross-sectional area of the psoas major muscle at the third lumbar vertebral level, correlates well with skeletal muscle mass index and is versatile because of its simplicity [52]. However, there are currently no established cut-off values for PMI. Because the average muscle mass differs depending on race and age, it is necessary to establish muscle mass cut-off values by conducting large-scale studies with respect to regions.

This study has some limitations. The use of the PMI marker in this study was a simple method, but it is necessary to clarify the degree to which PMI correlates with total body muscle mass in a large-scale clinical trial. In addition, the current trend in the evaluation of sarcopenia is to measure not only muscle mass but also intramuscular adipose tissue content and to evaluate muscle quality as well [53]. The diagnostic criteria for sarcopenia [48, 54] also incorporate items such as muscle strength and physical function. However, since patients who meet the current diagnostic criteria for sarcopenia are often judged to be inoperable to begin with and are not indicated for surgery, this study focused only on the PMI value as an indicator of low muscle mass for comparison.

## Conclusion

Preoperative PMI is an independent risk factor for distal cholangiocarcinoma recurrence after PD. Distal cholangiocarcinoma patients with low PMI may be more prone to recurrence and have a poorer prognosis.

## Supplementary Information

**Additional file 1: Table 1.** Characteristics of patients undergoing PD for distal cholangiocarcinoma.

**Additional file 2: Table 2.** Characteristics of patients in the early recurrence and non-early recurrence groups.

**Additional file 3: Table 3.** Comparison between the low and high PMI groups based on cut-off values.

**Additional file 4: Table 4.** Univariate and multivariate analyses of predictive factors using logistic regression analysis.

## Data Availability

The data that support the findings of this study are not publicly available due to privacy or ethical restrictions, but are available from the corresponding author upon reasonable request.

## Abbreviations

CI: Confidence interval
CRP: C-reactive protein
CT: Computed tomography
IQR: Interquartile range
mGPS: modified Glasgow Prognostic Score
MRI: Magnetic resonance imaging
NLR: Neutrophil-to-lymphocyte ratio
OR: Odds ratio
PD: Pancreatoduodenectomy
PLR: Platelet-to-lymphocyte ratio
PMI: Psoas muscle mass index
PNI: Prognostic nutritional index
RFS: Recurrence-free survival
ROC: Receiver operating characteristic
SMI: Skeletal muscle mass index
