# Correction: Low preoperative psoas muscle mass index is a risk factor for distal cholangiocarcinoma recurrence after pancreatoduodenectomy: a retrospective analysis

**DOI:** 10.1186/s12957-022-02882-x

**Published:** 2022-12-28

**Authors:** Saori Umezawa, Shinjiro Kobayashi, Takehito Otsubo

**Affiliations:** Division of Gastroenterological and General Surgery, St. Marianna University School of Medicine, 2-16-1 Sugao, Miyamae-ku, Kawasaki, Kanagawa 216-8511 Japan

**Correction: World J Surg Oncol 20, 176 (2022)**

**https://doi.org/10.1186/s12957-022-02627-w**

Following the publication of the original article [1], the author reported and error in the maintext. Second line in the left column of Page 5, “recipients” needs to be “donors”. The whole sentence should read

“These values approximate the cut-of values (6.36 cm^2^ /m^2^ for males and 3.92 cm^2^ /m^2^ for females) that defined the low muscle mass of 541 Japanese living donor liver transplant donors in previous studies [25, 26].”

The original article has been updated.
