# Supplementary material for: Low preoperative psoas muscle mass index is a risk factor for distal cholangiocarcinoma recurrence after pancreatoduodenectomy: a retrospective analysis
Source: World J Surg Oncol. 2022 Jun 2;20:176. doi: 10.1186/s12957-022-02627-w (PMC9161607; doi:10.1186/s12957-022-02627-w)
Supplement: Supplementary file 2 — Additional file 2: Table 2. Characteristics of patients in the early recurrence and non-early recurrence groups. [file 12957_2022_2627_MOESM2_ESM.pptx]

## Slide 1
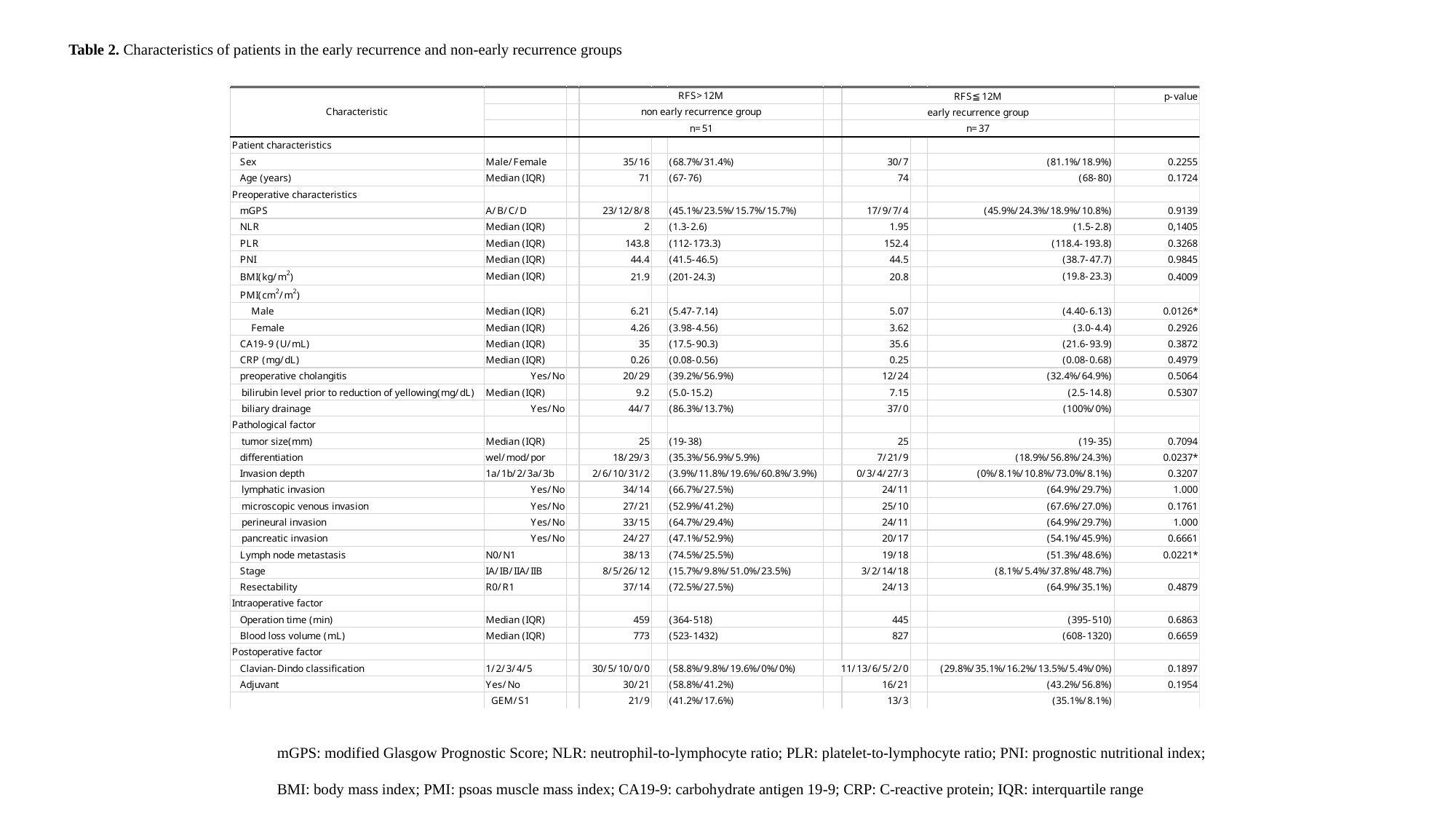

Table 2. Characteristics of patients in the early recurrence and non-early recurrence groups
mGPS: modified Glasgow Prognostic Score; NLR: neutrophil-to-lymphocyte ratio; PLR: platelet-to-lymphocyte ratio; PNI: prognostic nutritional index; BMI: body mass index; PMI: psoas muscle mass index; CA19-9: carbohydrate antigen 19-9; CRP: C-reactive protein; IQR: interquartile range
