# Supplementary material for: Low preoperative psoas muscle mass index is a risk factor for distal cholangiocarcinoma recurrence after pancreatoduodenectomy: a retrospective analysis
Source: World J Surg Oncol. 2022 Jun 2;20:176. doi: 10.1186/s12957-022-02627-w (PMC9161607; doi:10.1186/s12957-022-02627-w)
Supplement: Supplementary file 4 — Additional file 4: Table 4. Univariate and multivariate analyses of predictive factors using logistic regression analysis. [file 12957_2022_2627_MOESM4_ESM.pptx]

## Slide 1
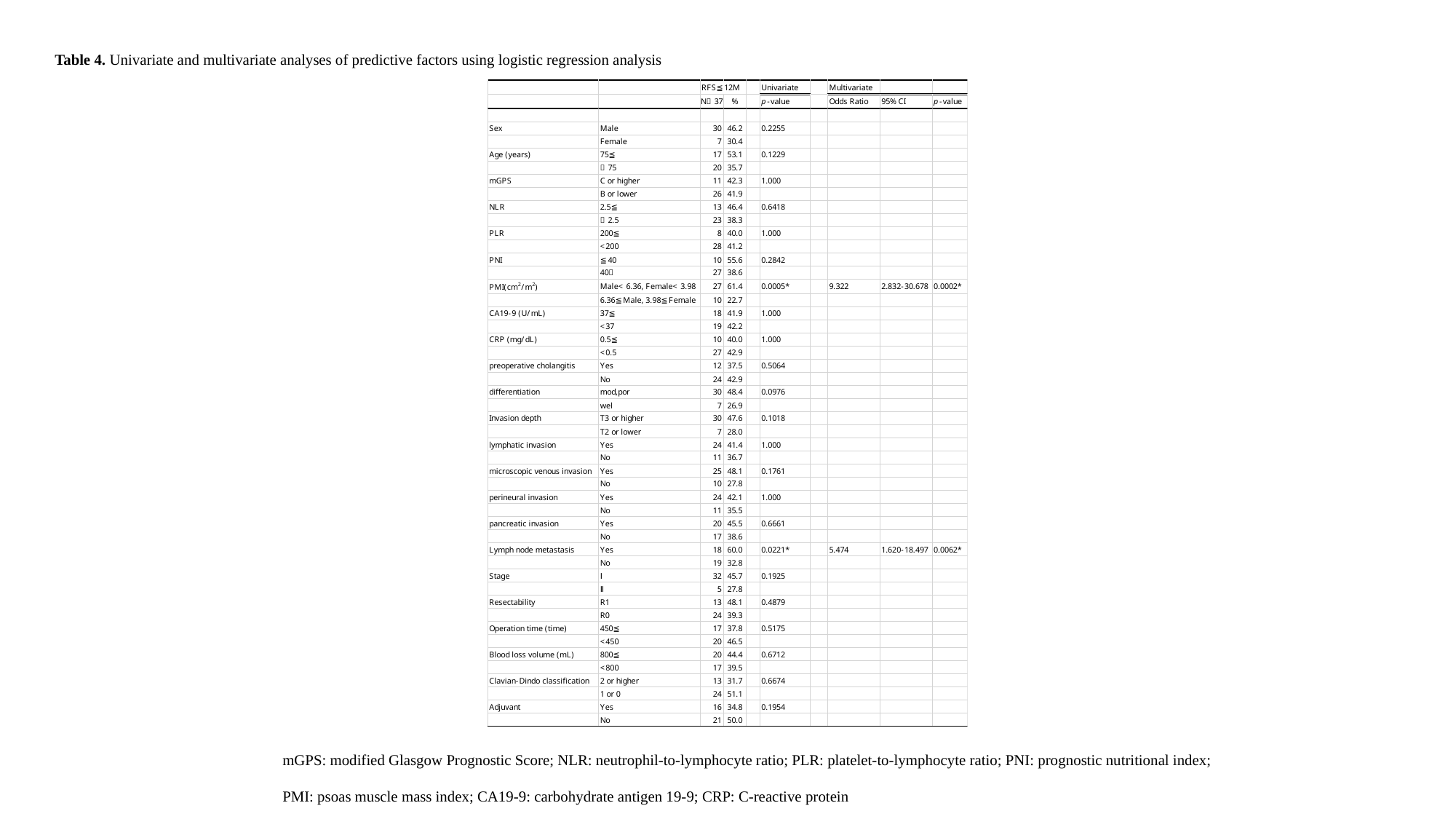

Table 4. Univariate and multivariate analyses of predictive factors using logistic regression analysis
mGPS: modified Glasgow Prognostic Score; NLR: neutrophil-to-lymphocyte ratio; PLR: platelet-to-lymphocyte ratio; PNI: prognostic nutritional index; PMI: psoas muscle mass index; CA19-9: carbohydrate antigen 19-9; CRP: C-reactive protein
